# Supplementary figures and images for: Identification of Candidate Genes Related to the Husk Papillae in Foxtail Millet (Setaria italica (L.) P. Beauv)
Source: Plants (Basel). 2025 Aug 14;14(16):2535. doi: 10.3390/plants14162535 (PMC12389439; doi:10.3390/plants14162535)

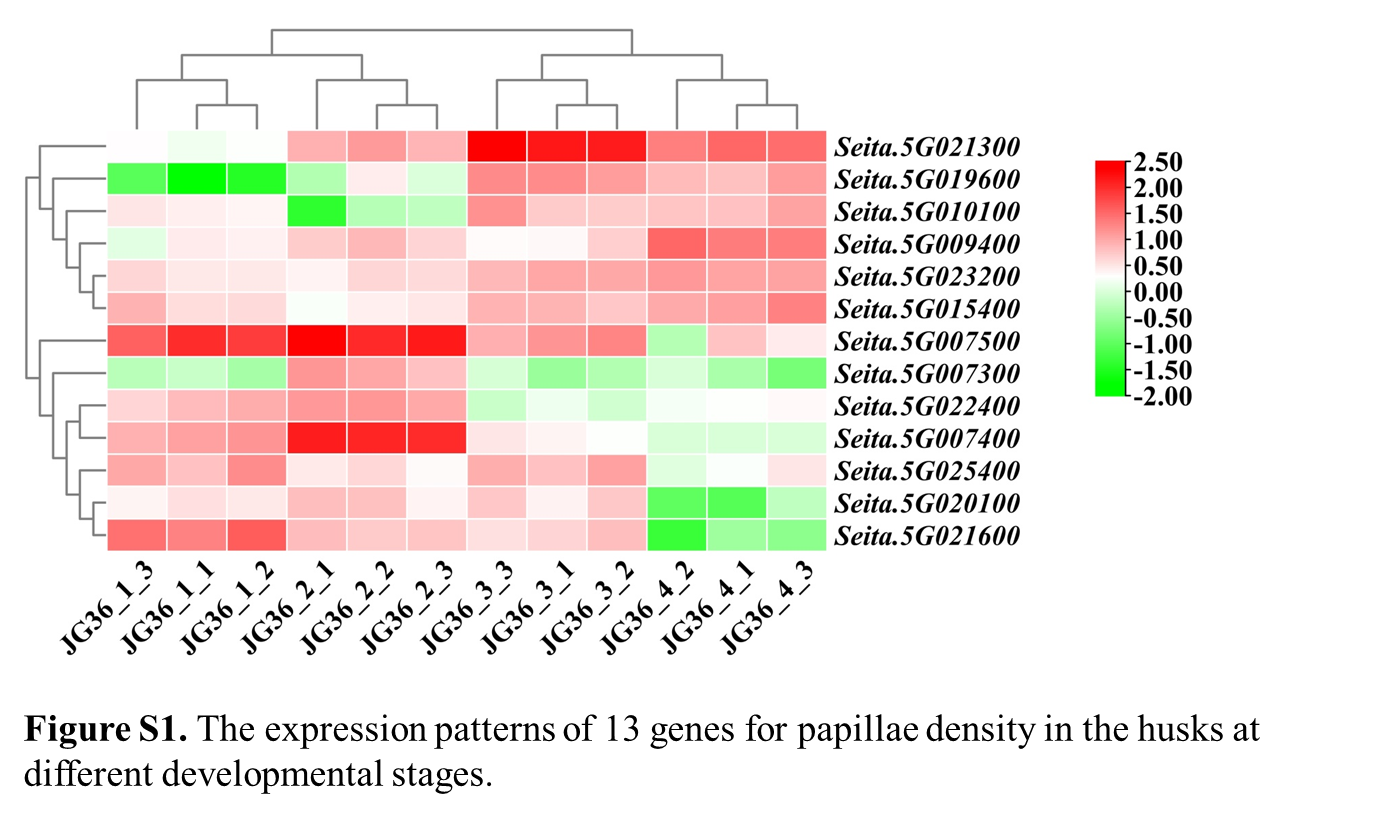

Supplement: Supplementary file 1 [file plants-14-02535-s001.zip › Figure S1.png]
